# Effectiveness of a Problem-Solving Program in Improving Problem-Solving Ability and Glycemic Control for Diabetics with Hypoglycemia

**DOI:** 10.3390/ijerph18189559

**Published:** 2021-09-10

**Authors:** Fei-Ling Wu, Chia-Hung Lin, Chia-Ling Lin, Jyuhn-Huarng Juang

**Affiliations:** 1 Department of Nursing, Chang Gung University of Science and Technology, Taoyuan 333, Taiwan; flwu@mail.cgust.edu.tw (F.-L.W.); lcling@mail.cgust.edu.tw (C.-L.L.); 2 Division of Endocrinology and Metabolism, Department of Internal Medicine, Chang Gung Memorial Hospital, Taoyuan 333, Taiwan; adronlin@cgmh.org.tw; 3 Department of Chinese Medicine, College of Medicine, Chang Gung University, Taoyuan 333, Taiwan; 4 Department of Medicine, College of Medicine, Chang Gung University, Taoyuan 333, Taiwan

**Keywords:** diabetes, problem-solving, hypoglycemia, self-management, glycated hemoglobin

## Abstract

The purpose of this study was to evaluate the effects of a hypoglycemia problem-solving program (HPSP) on problem-solving ability and glycemic control in diabetics with hypoglycemia. This was a prospective, quasi-experimental study with two groups, using a pre- and post-repeated measures design. A total of 71 diabetic patients with hypoglycemia were purposively assigned to an experimental group (*n* = 34) and a control group (*n* = 37). The experimental group participated in an 8-week HPSP, and each weekly session lasted approximately 90 min, while the control group received usual care. Participants were assessed at baseline, 1, 3, and 6 months after intervention care. In the experimental group, 6 months after the HPSP intervention, HbA1c was superior to that before the intervention. In both groups, the score obtained using the hypoglycemia problem-solving scale (HPSS) was low before the intervention. In the experimental group, HPSS tracking improved at all stages after the intervention compared to before the intervention. In the control group, the HPSS score improved slightly in the first month and sixth months after usual care. There were significant differences between and within groups in HbA1c levels and HPSS score over time. The intervention based on the HPSP effectively improves HbA1c level and hypoglycemia problem-solving ability in patients with hypoglycemia.

## 1. Introduction

In 2016, the World Health Organization issued a global call for action on dedicating greater attention to diabetes and identifying relevant solutions [1]. Although diabetes-related medical treatments and health care in Taiwan continue to improve, a survey on type 2 diabetes in 2018 revealed that 2.2 million people in Taiwan had diabetes, with the prevalence being 9.32%, and the number of new patients being nearly 160,000 annually. Because of the continual increase in the number of patients with diabetes, the pursuit of optimal glycemic control and reduction of possible complications has long been a major goal in diabetes care [2]. Therefore, patients with poor glycemic control should be encouraged to adopt insulin regimens as early as possible [3,4]. According to statistics from the National Health Insurance (NHI) database in Taiwan, the use of insulin regimens increased annually until 2014, with 4.67% of people with diabetes using insulin injections alone, and 8.01% using concomitant oral agents and insulin [5]. A global survey of patients receiving insulin treatment indicated that the incidence of hypoglycemia is as high as 78.3% [6]. The cost of achieving effective glycemic control may increase the incidence of hypoglycemia. As patients learn to adapt to having diabetes, they face a typical predicament: they are concerned about experiencing hypoglycemia but are also aware that addressing the symptoms of hypoglycemia may lead to high blood sugar [7]. Moreover, recurrent hypoglycemic episodes tend to cause changes in nerve conduction between neuron synapses [8] as well as reduced intelligence [9]. Therefore, hypoglycemia has become a principal limiting factor in the management of patients with diabetes and may also lead to poor glycemic control in such patients [10].

The most effective method for preventing hypoglycemia is to provide guidance to patients with diabetes and their family and friends [11]. The current diabetes health education is based on a self-management model with the aim of maintaining optimal health and quality of life. Patients must learn specific decision-making, adjustment, and management methods according to their unique lifestyles, rather than allow the disease to control their living habits. Problem-solving is a logical thinking process [12] and it is the optimal approach for guiding patients to face care problems; moreover, an effective overall care ability can be gradually achieved through experience and learning [13]. When solving a problem, patients must use inductive reasoning and knowledge applications as they attempt to develop possible solution strategies. This process requires the participation of and flexible interactions between healthcare providers and patients [14]. Problem-solving has been demonstrated to be a critical ability for patients with diabetes to successfully master their self-management skills; however, it is also the most difficult skill for diabetes health educators to teach to patients [13]. In addition, the problem-solving ability of patients with hypoglycemia is positively correlated with their glycated hemoglobin (HbA1c) control status [15]. Problem-solving is affected by a patient’s own perspectives, personal factors, and external environmental factors. Thus, health educators must first understand a patient’s solution and evaluate their disease problems before providing individual guidance skills to help the patient effectively implement self-management [16].

Scholars point out that in the face of long-term and unchangeable health problems, they may lack self-efficacy for problem solving, which often affects the application of problem-solving skills [17]. According to research on diabetes care, problem-solving interventions not only improve patients’ problem-solving ability but also have a positive impact on the physical and psychological results of disease care [18,19]. Patients’ learning of effective problem-solving methods and their use of blood glucose self-monitoring data enable them to provide effective explanations, make informed decisions, accurately adjust their insulin dosage, calculate the composition of their foods, and perceive the significance of and risks indicated by the data [16]. Our goal is to coach the participants’ problem-solving skills to enhance self-efficacy in identifying symptoms, analyzing the causes and devising strategies to solve them, and effectively achieve glycemic control. Therefore, the purpose of this study was to evaluate the effectiveness of a problem-solving intervention program in the self-management of hypoglycemia in patients with diabetes.

## 2. Materials and Methods

### 2.1. Participants and Sample Size

Purposive sampling was used to recruit patients with diabetes from the metabolic outpatient clinics of two major medical centers and regional hospitals. Healthcare providers referred outpatients who satisfied the inclusion criteria. These patients were then invited by the researchers to participate in the study. The inclusion criteria were as follows: (1) being ≥20 years of age, (2) living with diagnosed type 2 diabetes for at least 1 year, (3) having experienced at least one hypoglycemic episode in the previous 6 months (hypoglycemic episode: blood glucose level <70 mg/dL or presenting hypoglycemic symptoms of shaking, sweating, drowsiness, or behavioral changes), and (4) being treated with insulin. Patients were excluded if they had renal failure, mental illness, blindness, severe physical handicap or if they were pregnant.

G-power version 3.1 was adopted to estimate the appropriate sample size, which was 64 participants; considering a 20% attrition rate, the required sample size was 76. The corresponding statistical parameters were as follows: α = 0.05, power = 0.80, covariance R^2^, and effect size = 0.40. This study was approved by the institutional review board of a medical center in northern Taiwan (1040147B). All participants provided written informed consent before data collection.

### 2.2. Study Design

This was a prospective, quasi-experimental study with two groups, and a pre- and post-test repeated-measures design was employed. In order to avoid interventional contamination, a draw was used to determine patients with single-month admissions as the experimental group and patients with bimonthly admissions as the control group. Participants in the experimental group participated in the hypoglycemic problem-solving program (HPSP), whereas the control group received usual care. The main aim was to compare the frequency of hypoglycemia, HbA1c level, and hypoglycemic problem-solving abilities between the experimental and the control groups.

### 2.3. Interventions

The HPSP was developed on the basis of the theory of social problem-solving. The program consisted of two phases with a total duration of 8 weeks, and the weekly activity time was approximately 90 min. The goal of the activities during the first stage was to learn about problem-solving. The diabetes team (consisting of a physician, a health educator, and a nutritionist) provided instruction on basic hypoglycemia care to improve the participants’ accuracy in solving hypoglycemia problems. Phase 2 focused on the application of problem-solving skills. We used situational case introduction, group discussions, experience sharing, and individual guidance to summarize the causes of hypoglycemia, develop feasible goals and strategies, and perform self-analysis on the reasons for the difficulty or success of the implemented strategy as a reference for the self-management of hypoglycemia. Table 1 provides a description summarizes the details of the program. Before each weekly activity, the study team sent reminders to the participants by phone. The comparison group received usual care only. Health education nurses provided instruction in the hypoglycemia education leaflet (identification of hypoglycemia, management of hypoglycemia, and prevention methods).

Baseline data for all measures were obtained before intervention assignment. The participants were assessed at four time points: the baseline assessment (T0) was conducted before the HPSP, with follow-up assessments 1 (T1), 3 (T2), and 6 (T3) months after participation in the HPSP. Outcome measures were HbA1c and Hypoglycemia Problem-Solving Scale (HPSS) scores.

### 2.4. Research Instruments

#### 2.4.1. Demographic and Disease Characteristics

The demographic variables were sex, age, marital status, educational level, and employment status. Disease characteristics were medication regimen, duration of insulin treatment, and frequency of hypoglycemic episodes in the previous 6 months. HbA1c was measured at the beginning of the study and at the 1-, 3-, and 6-month follow-ups. Demographic and disease-related characteristics were obtained from chart reviews and self-monitored blood glucose records.

#### 2.4.2. HPSS

The HPSS was used to measure the participants’ problem-solving ability in terms of hypoglycemia [20]. The HPSS consists of 24 items scored on a 5-point Likert scale from 0 (not applicable) to 4 (always applicable), and higher scores indicate higher degrees of problem-solving ability for hypoglycemia. The Cronbach’s alphas of the HPSS ranged from 0.83 to 0.90 in this study, indicating favorable to excellent internal consistency reliability.

### 2.5. Statistical Analysis

SPSS, version 23.0 for Windows (Armonk, NY, USA) was adopted to analyze the data. Descriptive statistical analysis, including mean (*SD*), counts, and percentages, was employed to describe the demographic data, disease characteristics, and hypoglycemia problem-solving ability. Inferential statistical analysis was performed using a chi-square distribution test, *t* test, and Fisher’s exact test. The effects of the HPSP on frequency of hypoglycemic episodes, HbA1c levels, and hypoglycemic problem-solving ability were analyzed using a repeated-measures analysis of variance (ANOVA), including the assessment of changes over time and the time × group interaction effect.

## 3. Results

### 3.1. Participant Characteristics by Group

**Figure 1:**
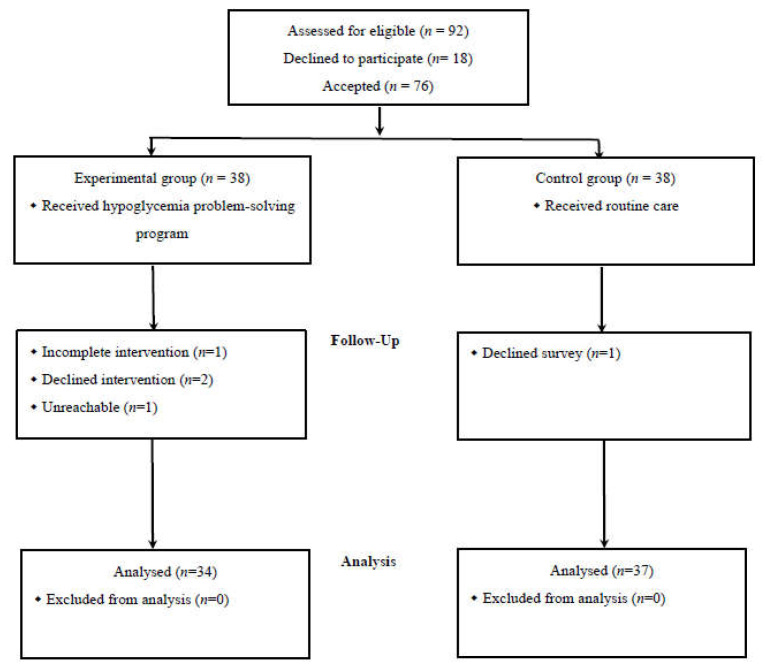
Recruitment for the Hypoglycemia Problem Solving Program.

A total of 76 eligible patients were invited to participate in this study. Five participants withdrew during the intervention process because of limited time availability, unwillingness to participate, and transportation problems. Therefore, the data of 71 participants were analyzed; the experimental and control groups included 34 and 37 participants, respectively (Figure 1). The intervention completion rate was 93.4%. To ensure the accuracy of the experimental results, a chi-square test and an independent sample *t* test were employed to assess the homogeneity of the two groups. No significant differences were observed between the two groups (*p* > 0.05) regarding demographic or disease characteristics (Table 2). The participants in the experimental and control groups had average ages of 53.71 (*SD* = 15.66) and 54.87 (*SD* = 15.28) years, respectively. More than half of the participants were men, married, and working, and the most common educational level was senior high school. In addition, 70.4% of the participants’ medication regimen was insulin injections alone. The mean duration of the insulin treatments was 7.19 (*SD* = 7.24) years. In the preceding 6 months, 69.01% of the participants had experienced ≥6 hypoglycemic episodes.

### 3.2. Comparison of Group Outcomes at Baseline

**Figure 2:**
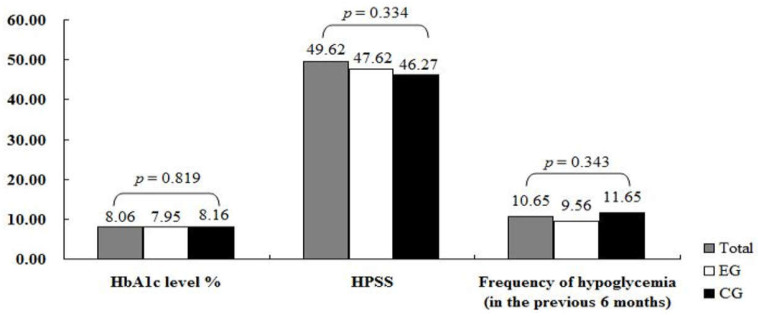
Comparison of group outcomes at baseline (N = 71): EG, experimental group (*n* = 34); CG, control group (*n* = 37); HPSS, Hypoglycemia Problem-Solving Scale.

The HbA1c, HPSS, and hypoglycemic episodes results for the experimental and control groups at baseline are presented in Figure 2. At baseline, no significant differences were observed between the two groups in HbA1c level (*Z* = −0.053, *p* > 0.05), HPSS (*Z* = −0.948, *p* > 0.05), and frequency of hypoglycemia (*t* = −0.913, *p* > 0.05).

### 3.3. Effects of the HPSP on HbA1c Levels and HPSS Scores

The results of the repeated-measures ANOVA for HbA1c levels, HPSS scores, and hypoglycemic episodes of the two groups at the four evaluation times are listed in Table 3. Regarding the HbA1c results, in the experimental group (HPSP), the average HbA1c level from T1 (1 month after intervention) to T3 (6 months after intervention) was significantly lower than that at T0 (before intervention). The average HbA1c levels from T0 to T1 and T2 to T3 decreased significantly, whereas that from T1 to T2 rose slightly; moreover, the average HbA1c level at T3 was significantly lower than that at T0, and HbA1c reached a statistically significant difference over time (*F* = 5.090, *p* < 0.01). The HbA1c levels of the control group (usual care) did not change significantly from T0 to T3. HbA1c at T3 was not significantly higher than that at T0. The average total HPSS score of the two groups before the intervention was less than half of the total potential score (47.62 vs. 46.27 points). After the intervention, the HPSS scores of the experimental group from T1 to T3 were significantly higher than that at T0, and the average problem-solving ability scores from T0 to T1 and T2 to T3 increased significantly, whereas that from T1 to T2 decreased slightly. In the control group, the average HPSS scores at T1 and T3 were higher than that at T0, and the two groups differed significantly in HPSS scores. There was no significant change in the frequency of hypoglycemia from T0 to T3 in the control and experimental groups. However, the frequency of hypoglycemia in the experimental group showed a decreasing trend from T2 to T3.

The two-way repeated-measures ANOVA revealed a significant interaction of time and group for HbA1c level after the intervention (*F* = 5.816, *p* < 0.01). After 6 months, the reduction of HbA1c in the experimental group was significantly greater than in the control group. Notably, no significant difference was observed between the two groups of patients with diabetes in terms of their ability to resolve hypoglycemia problems before the start of the program. After 8 weeks of HPSP intervention and a 6-month interval, the hypoglycemia problem-solving ability at T3 (6 months) in both groups was higher than that at T0 (baseline). Although the problem-solving ability of the control group (usual care) improved slightly, the effectiveness of this ability was significantly lower that of the experimental group (*F* = 13.653, *p* < 0.001; Table 3).

## 4. Discussion

Diabetes is a chronic disease that requires patients to implement self-management; therefore, health education strategies are instrumental in disease treatment and blood sugar control [21]. Although there is a rich body of research on the effects and management of diabetes, most studies focus on improving knowledge and behaviors in self-management [22,23]. The literature is sparse in relation to the implementation of protocols to manage hypoglycemia. Problem-solving skills are required for education and cumulative experience training [13]. Our program facilitates healthcare providers’ work with patients to develop care goals and plans and provides problem-solving skills using a variety of strategies to address the realities of caring for people with hypoglycemia. The training provided in the HPSP in this study focused on problem-solving guidance. The HPSP intervention emphasizes that health-care personnel must guide patients with type 2 diabetes to clarify the cause of hypoglycemic events that they experience and must help patients learn to self-monitor their blood glucose; self-monitoring helps patients detect the factors of hypoglycemia and make timely corrections in their disease self-management according to the monitored values. The healthcare personnel’s assistance with adjusting patients’ daily treatment methods can reduce the patients’ sense of frustration during problem-solving. Therefore, educational programs regarding hypoglycemia events should focus on understanding the problems and analyzing the possible causes of hypoglycemia.

The HPSP intervention had a significant effect on HbA1c levels, with values from T1 to T3 being lower than that at T0 (7.95%) before the intervention. Because a longer period had elapsed after the intervention, the HbA1c level at T2 (7.63%) was higher than that at T1 (7.41%). This project emphasized patient autonomy in disease care and focused on the learning and application of problem-solving, including the numerous aspects that must be considered in hypoglycemia self-management. After a period of dedication, the participants’ perception of hypoglycemia might have been reduced. Similarly, their ability to solve hypoglycemia problems at T2 (58.59 points) was slightly lower than that at T1 (60.65 points). Thus, HbA1c control may have been affected by the participants’ problem-solving ability. Studies have suggested that training in problem-solving requires patients and healthcare personnel to act as partners in a diabetes care team; this approach helps cultivate patients’ problem-solving skills to manage diverse life situations [24,25]. The results of the current 6-month short-term follow-up indicated that the participants interacted with the care team through blood glucose monitoring and were taught how to interpret their monitoring results and improve their blood glucose on the basis of the measured values. Frequent discussions and reviews of the strategies not only improved the participants’ ability to solve hypoglycemia problems but also supported their blood sugar control, especially for participants with hypoglycemia who actively maintained optimal blood sugar control. In addition, Schmitt et al. showed that better self-management was associated with better control of HbA1c levels [26]. The mean HbA1c of the participants in this study was 8.06%; although there was no significant difference between the samples of the two groups before the intervention, their previous experience may have influenced their self-management and compliance to control HbA1c levels.

The HPSP intervention had a significant effect on HPSS scores. In this study, the average HPSS score of the two groups before the intervention was less than half of the total score (full score of 96) and lower than the average HPSS score (58.22) recorded in a study investigating hypoglycemia in patients with type 1 and type 2 diabetes [15]. This result indicates that the participants in the present study were generally deficient in their problem-solving ability for hypoglycemia, which might be related to the long-term nature of higher blood glucose in patients with type 2 diabetes; that is, these patients no longer regard hypoglycemia events as a challenge requiring aggressive treatment. The HPSS score in the experimental group (HPSP intervention) increased significantly by nearly 20 points from T0 to T3. This result indicates that in the HPSP intervention, the healthcare team helped the participants to develop problem-solving skills, which improved the participants’ positive thinking and awareness of hypoglycemia, in addition to enhancing their willingness to participate in problem-solving and their belief that hypoglycemia can be resolved.

The control group received only conventional health education, and although their scores after the intervention differed significantly from the baseline scores, the scores for hypoglycemia problem-solving ability at T3 (46.68) remained in the middle or lower level. The results suggest that, although the provision of conventional health education through medical and nursing personnel improved the participants’ problem-solving ability or compliance with health education, addressing hypoglycemia-related problems requires a clear goal that is developed according to patients’ daily information. This phenomenon might also be related to the fact that the participants in this study were mainly middle-aged and older patients with type 2 diabetes. Thus, patients in these age groups require thorough educational interventions from health-care personnel, which should include more interactive learning opportunities to compensate for the learning obstacles caused by aging. Although the HPSP intervention had no significant effect on hypoglycemic episodes, the frequency of hypoglycemia in the experimental group showed a decreasing trend at T3 compared to T2. This is a limit of our study, related to the different collection times between the T0 and the T3 stages, and the study design should be improved in the future.

Patients with diabetes face diverse challenges that are affected by several personal and environmental factors. Scholars have suggested that patients should learn to adjust their lifestyles according to their particular situations. Moreover, problem-solving is a critical element in self-management [27], and its effect on healthcare behaviors should not be neglected. This study verified that a problem-solving intervention could significantly improve the hypoglycemia care of patients with diabetes.

### Limitations and Suggestions

Several limitations of our study should be considered. First, many relevant factors were not included in the analysis, such as the evolution of the pharmacological therapy, body mass index, physical exercise, meal times, consumption of snacks, and stress, which may result in residual confounding. Second, time limitation affects the observation effect. The intervention period of this study was six months. To understand whether the participants could show the behavioral effect in the long term, it is suggested that a longer intervention period should be observed in the future. Third, the management of hypoglycemia is a core learning component of sick day management, and future research suggests that it be included in the guidelines.

## 5. Conclusions

The current HPSP intervention effectively improved the problem-solving abilities of the experimental group with hypoglycemia as measured by HbA1c level and HPSS scores. Although conventional health education did not have a significant effect on the HbA1c level of the control group, it had a small significant effect on problem-solving ability for hypoglycemia at 6 months after the intervention compared to problem-solving ability before the intervention. Problem-solving ability training involves the accumulation of experiences and learning and emphasizes the importance of interactions between healthcare providers and patients. For effective hypoglycemia-related problem-solving, patients must apply their knowledge, analyze the causes of hypoglycemia, and attempt to develop their own solution strategies, so to maintain an ideal blood sugar level and reduce the frequency of hypoglycemia. The current results may serve as a reference for future hypoglycemia care plans.

## Figures and Tables

**Table 1:** Course Schedule for the HPSP.

Stage Number/Main Topic	Weekly	Course Content
Stage1 Recognition problem-solving	1–2 w	Learn the steps and skills of problem-solving 1. problem orientation responses 2. problem-solving perception 3. detection control 4. problem-solving skill 5. problem-solving principles
	3–4 w	learned about hypoglycemia and self-care 1. blood glucose for self-monitoring 2. identify the symptoms and signs of hypoglycemia 3. hypoglycemia problem definition and formulation 4. analyze the causes of hypoglycemia 5. generation of alternative solutions
Stage2 Application of problem-solving skills	5–6 w	clarify hypoglycemia-related problems problem-solving health education manual, case studies and discussions, situational case introduction, group discussions, and experience sharing (diet, exercise, medication, body weight control, disease stress adjustment, and blood glucose self-monitoring)
	7–8 w	implement and evaluate the solution strategies 1. coaching, modeling, shaping, rehearsal 2. solution implementation 3. verification

**Table 2:** Homogeneity test of the demographic and disease characteristics of the two groups (N = 71).

Variables	Total (N = 71)		Experimental Group (*n* = 34)		Control Group (*n* = 37)		*χ^2^/t*	*p*
	*N*%/*M* ± *SD*		*n*%/*M* ± *SD*		*n*%/*M* ± *SD*			
Demographic data								
Gender							0.104	0.814
Male	39	54.93	18	52.9	21	56.8		
Female	32	45.07	16	47.1	16	43.2		
Age (years)	53.10 ± 15.98		53.71 ± 15.66		54.87 ± 15.28		0.158	0.692 ^a^
≤40	18	25.4	7	20.6	11	29.7		
41–50	13	18.3	9	26.5	4	10.8		
51–60	12	16.9	3	8.8	9	24.3		
61–70	16	22.5	9	26.5	7	18.9		
>70	12	16.9	6	17.6	6	16.3		
Marital status							0.592	0.502
Unmarried	24	33.8	11	32.3	13	35.1		
Married	47	66.2	23	67.7	24	64.9		
Education level							3.320	0.851 ^a^
Junior high school or below	16	22.5	7	20.6	9	24.3		
Senior high school	31	43.7	16	47.1	15	40.6		
University or above	24	33.8	11	32.3	13	35.1		
Employment status							3.267	0.073
Unemployed	20	28.2	13	38.2	7	18.9		
Working	51	71.8	21	61.8	30	81.1		
Disease characteristics							0.012	0.963
Medication regimen								
Insulin	50	70.4	24	70.6	26	70.3		
Oral medication and Insulin	21	29.6	10	29.4	11	29.7		
Duration of insulin treatment (years)	7.19 ± 7.24		7.26 ± 7.45		7.11 ± 7.15		0.474	0.946
≤5	41	57.7	19	55.9	22	59.4		
≥5.1	30	42.3	15	44.1	15	40.6		
Frequency of hypoglycemia								
(in the previous 6 months)	10.65 ± 8.38		9.56 ± 7.91		11.65 ± 8.78		0.913	0.343
<6	22	31.0	11	32.3	11	29.7		
≥6	49	69.0	23	67.7	26	70.3		

^a^ Fisher’s exact test.

**Table 3:** Repeated-measures analysis results for HbA1c level and HPSS scores by group (N = 71).

Variables	Group	T0 Mean *(SD)*	T1 Mean *(SD)*	T2 Mean *(SD)*	T3 Mean *(SD)*	Time *F (p)*		Time * Group *F (p)*	
HbA1c	EG	7.95 (1.20)	7.41 (0.98)	7.63 (0.84)	7.22 (0.61)	5.090	<0.01	5.816	<0.01
	CG	8.16 (1.30)	8.20 (0.58)	8.13 (1.04)	8.30 (1.03)	2.487	0.080		
HPSS	EG	47.62 (14.71)	60.65 (9.29)	58.59 (8.66)	64.53 (9.73)	19.476	<0.001	13.653	<0.001
	CG	46.27 (12.52)	46.78 (11.81)	46.05 (11.80)	46.68 (11.67)	5.113	<0.05		
FOH	EG	9.56 (7.91)	1.51 (1.08)	4.08 (1.78)	3.24 (0.75)	2.910	0.068	3.114	0.073
	CG	11.65 (8.78)	1.96 (1.45)	5.34 (2.11)	6.63 (0.83)	0.872	0.640		

EG, experimental group; CG, control group; FOH, frequency of hypoglycemia; HPSS, Hypoglycemia Problem-Solving Scale; T0, baseline; T1, 1 month after HPSP or usual care; T2, 3 months after HPSP or usual care; T3, 6 months after HPSP or usual care. HPSP, Hypoglycemia Problem-Solving Program. Time * Group, F value of the interaction of between groups and within pre- and post-test.

## Data Availability

Data are from 71 participants of the metabolic outpatient clinics of two major medical centers and regional hospitals. Due to legal restrictions imposed by the government of Taiwan in relation to the “Personal Information Protection Act”, data cannot be made publicly shared.
